# Supplementary material for: Physical activity and diet pattern do not mitigate C-reactive protein increases associated with oral contraceptive use
Source: PLoS One. 2025 Apr 8;20(4):e0319928. doi: 10.1371/journal.pone.0319928 (PMC11978021; doi:10.1371/journal.pone.0319928)
Supplement: Attachment — Submitted filename: Response to Reviewers.docx [file pone.0319928.s001.docx]

Response to Reviewers

PONE-D-24-18364

Physical activity and diet pattern do not mitigate C-reactive protein increases associated with oral contraceptive use

We would like to thank the editor and both reviewers for their insight and constructive feedback. We have addressed all reviewer comments with revisions that have strengthened the manuscript. Please find responses to all reviewer comments below.

**Reviewer #1**
1) Line 87-89: I felt that the sentence about possible mechanisms that could increase CRP levels in OCP users could do with some work. In this sentence 'but' could possibly be replaced with 'of which'.

We agree that this sentence lacked clarity in its original form. Thank you for bringing this to our attention. The statement now reads:

“There are several mechanisms by which OCs could increase CRP [16], which may include both systemic inflammation and localized effects on hepatic production of CRP.”

2) Line 276: should 'oral contraceptives' be presented as OC as per the rest of the article?

You are correct. We have updated the manuscript to ensure that “oral contraceptive” is abbreviated to “OC” in all instances following its first use (except at the beginning of new sentences, per common grammatical norms).

**Reviewer #2**
1) It is stated that it is unclear “if these elevations [in CRP] can be mitigated or attenuated by physical activity, low BMI, or anti-inflammatory diet patterns”, yet you have discussed a study (Larsen et al 2020) showing significantly elevated CRP levels even in Olympic athletes with a low BMI (19.5), which seems to suggest that a low-normal BMI and high levels of PA will not attenuate CRP levels in OC users – this finding (also observed in other studies) needs further consideration/discussion in the Introduction to add clarity surrounding the aims and hypotheses of this research

Thank you for highlighting this lack of clarity. The introduction has been revised as follows:

“Even among world-class endurance and team sport athletes, CRP levels are significantly elevated in OC users [15]. It is possible that acute stressors of intense training could contribute to these observed CRP elevations, but direct comparisons to OC nonusers engaged in similar training cast doubt on this potential explanation [15]. Preliminary studies appear to suggest that OC users are unable to fully mitigate elevations in CRP by maintaining low adiposity and high PA levels, but these observations are based on very few studies with relatively small sample sizes and homogenous populations, such as university students or athletic teams.”

“There are currently several unresolved questions pertaining to the clinical implications of CRP elevations induced by OC use. Most notably, it is unclear if OC-induced elevations in CRP reflect a systemic inflammatory response and if these elevations can be mitigated or attenuated by physical activity, low BMI, or anti-inflammatory diet patterns in the general population.”

2) Line 128: were all OC users included? What about those on progestin-only pills? Was length of OC use captured?

Thank you for prompting us to clarify these points. The manuscript now contains the following statements:

“The NHANES data do not distinguish between combined OCs (with synthetic estrogen and progestin) and progestin-only pills, so the present analysis includes all OC users. However, the inclusion of progestin-only pill users is unlikely to substantially impact the present analysis because less than 1% of US women use progestin-only pills [22].” (in methods section)

“The NHANES data do not distinguish between combined OCs and progestin-only pills, although this impact is likely negligible as less than 1% of US women use progestin-only pills [22].” (in limitations section)

“Survey questions about OC use specifically refer to current use, so duration of OC use is unknown for survey respondents.” (in methods section)

“Surveys provided information about current OC use but did not provide details regarding history and duration of use.” (in limitations section)

3) Line 157: it is stated that “first generation oral contraceptives are rarely used in the present day” however data was captured ~20 years ago. Implications of this should be discussed as a limitation

Thank you for highlighting this consideration. The severe side effects associated with first-generation contraceptives were identified in the 1960s and 1970s, which led to rapid adjustments to estrogen doses and the introduction of second- and third-generation progestins. As a result, first-generation contraceptives were largely phased out of clinical practice by the time these NHANES data were collected in 1999-2006. The manuscript now contains the following statement:

“These NHANES questionnaires provide incomplete information regarding the generation of contraceptive used by each study participant. However, this is unlikely to impact the present study’s findings because first generation OCs were largely phased out of clinical practice by the 1990s and CRP elevations appear to be similar among second, third, and fourth generation OCs [20].”

4) Line 161: having a period every 2 months is not considered within the range of a regular MC. Thus, it is likely that subjects with MC dysfunction (and thus, a different hormonal profile to those with a regular MC) were captured in the study cohort. This also needs to be considered as a limitation

Our decision to use this criterion was largely driven by the specific survey item used in NHANES. The survey asks participants “When did you have your last period?” The first two options (reflecting the two most recent answer options) are “Having it now” and “Less than 2 months ago.” This likely reflects clinically pertinent observations that 1) many females may have “normal” menstrual cycles up to 35 days in length, and 2) missing an occasional menstrual cycle does not meet typical criteria for amenorrhea or oligomenorrhea. Nonetheless, this is an important consideration to highlight in our paper. The manuscript now contains the following statements:

“Menstrual status was assessed by item RHQ051 (“When did you have your last period?”). For the present analysis, participants were considered “regularly menstruating” if they selected “having it now” or “less than 2 months ago” for this survey item, as answers beyond 2 months are more likely to reflect clinically relevant menstrual cycle disorders.” (methods section)

“Similarly, we excluded OC nonusers who did not experience a menstrual cycle within 2 months of taking the NHANES survey, but it’s possible that a small number of participants with menstrual cycle disorders were included in the sample.” (limitations section)

5) Depending on responses to questions above, many different hormonal profiles could have been captured in the study i.e., those with regular MC, MC disorders, combined OC users, progestin only OC users… clarification and more detail is required around participant inclusion/exclusion criteria and the limitations thereof

We agree that these considerations are important to highlight in the manuscript. The limitations section now indicates:

“Surveys provided information about current OC use but did not provide details regarding history and duration of use. Similarly, we excluded OC nonusers who did not experience a menstrual cycle within 2 months of taking the NHANES survey, but it’s possible that a small number of participants with menstrual cycle disorders were included in the sample. The NHANES data do not distinguish between combined OCs and progestin-only pills, although this impact is likely negligible as less than 1% of US women use progestin-only pills [22]. These surveys do not identify the current menstrual cycle phase or distinguish between the active or inactive phase of OC pills at the time of each participant’s NHANES assessment, although is unlikely to introduce a substantive or systematic bias to our analysis [23]. The NHANES data also fail to specify the specific generation of OC being used by each participant, but CRP elevations are consistently observed across the generations that are currently used in clinical practice [20].”

6) Were CRP samples collected at a specific phase of the MC or OC use (active or inactive pills)?

Due to the large and cross-sectional nature of the NHANES study, it was not feasible to standardize participants’ MC phase or OC (active versus inactive) phase at the time of assessment. In addition, NHANES surveys do not capture this information. Fortunately this is unlikely to introduce systematic bias to our analysis, and previous literature indicates that CRP fluctuations across the menstrual cycle are small in magnitude and that OC-induced CRP elevations persist across all menstrual cycle phases and OC pill phases. We have added the following statements to our manuscript to reflect this information:

“Survey items related to OC use and menstrual status do not identify the current menstrual cycle phase or distinguish between the active or inactive phase of OC pills at the time of each participant’s NHANES assessment. However, this is unlikely to introduce systematic bias to our analysis, and previous literature indicates that CRP fluctuations across the menstrual cycle are small in magnitude and that OC-induced CRP elevations persist across all menstrual cycle phases and OC pill phases [23].” (in methods section)

“These surveys do not identify the current menstrual cycle phase or distinguish between the active or inactive phase of OC pills at the time of each participant’s NHANES assessment, although is unlikely to introduce a substantive or systematic bias to our analysis [23].” (in limitations section)

Discussion

7) Why are only 3 of the 5 hypothesis presented in Para 1 of the Discussion? The numbers don’t align to those previously used either (1-3 vs 1-5). This is confusing – please re-write to align discussion with other manuscript sections when discussion hypotheses 1-5

Thank you for this suggestion. We agree that the clarity of the paper would be improved by refining the numbering of hypotheses and maintaining consistency when discussing them in order. To eliminate redundancy from hypotheses with considerable overlap, we have condensed the list of hypotheses to 4, and these same 4 numbered hypotheses are discussed in order in the introduction section, the statistical analysis section, the results section, and the first paragraph of the discussion section. The manuscript has been revised accordingly.

The introduction section now reads:

“We hypothesized that 1) OC use and BMI would be positively associated with CRP levels in the full sample; 2) correlations between CRP and other indices of inflammation would be decoupled among OC users (reflecting OC-induced CRP elevations in the absence of a systemic inflammatory response); 3) PA level would only be associated with CRP levels among OC nonusers; and 4) DII would only be associated with CRP levels among OC nonusers. These hypotheses collectively reflect the perspective that OC-induced elevations in CRP are not indicative of systemic inflammation, and are therefore unresponsive to the anti-inflammatory effects of PA and low DII.”

The discussion section now reads:

“We hypothesized that 1) OC use and BMI would be positively associated with CRP levels in the full sample; 2) correlations between CRP and other indices of inflammation would be decoupled among OC users; 3) PA level would only be associated with CRP levels among OC nonusers; and 4) DII would only be associated with CRP levels among OC nonusers. Our findings lend support to hypotheses 1, 3, and 4, but contradict hypothesis 2. In line with previous research, CRP levels were positively associated with OC use and adiposity [29] but negatively associated with PA levels [12]. Contrary to our hypothesis, elevated CRP levels among OC users were correlated with alternative indices of inflammation and did not appear to be decoupled from systemic inflammation. Physical activity level, BMI, and DII values were significantly predictive of circulating CRP values among OC nonusers in this sample. Body mass index was significantly predictive of circulating CRP values among OC users in this sample, but PA level and DII were not. Collectively, these results suggest that OC-induced CRP elevations do appear to reflect systemic inflammation but are not meaningfully attenuated by physical activity or anti-inflammatory diet patterns.”
