# Supplementary material for: Physical activity and diet pattern do not mitigate C-reactive protein increases associated with oral contraceptive use
Source: PLoS One. 2025 Apr 8;20(4):e0319928. doi: 10.1371/journal.pone.0319928 (PMC11978021; doi:10.1371/journal.pone.0319928)
Supplement: Attachment — Submitted filename: Response_to_Reviewers_auresp_2.docx [file pone.0319928.s002.docx]

Response to Reviewers

PONE-D-24-18364R1

Physical activity and diet pattern do not mitigate C-reactive protein increases associated with oral contraceptive use

We would like to thank the editor and reviewers for their insight and constructive feedback. We have addressed all reviewer comments with revisions that have strengthened the manuscript. Please find responses to all reviewer comments below.

**Reviewer #1**
*Thank you to the authors for their edits to their paper. The changes have substantially improved the presentation of the work. I do have some additional comments that I would like the authors to consider and address prior to the paper being accepted for publication. I hope that the authors find these helpful.*

Thank you for your time, effort, and careful attention to detail throughout this review process. We believe this additional round of revisions has improved the manuscript even further.

1) *It is noted that the authors define the abbreviations in the abstract of the paper. However, in the introduction, the authors again define the abbreviations CPR and OC but do not redefine PA, BMI or DII. This may need to be reviewed.*

Thank you for highlighting this discrepancy. We believe it is helpful to define acronyms at first use in both the abstract and the full text, so in the interest of consistency we have updated the manuscript to define PA, BMI, and DII in the introduction section as well.

*2) Should line 86 state ‘that physical and body composition are insufficient for mitigating systemic inflammation’? The addition of body composition would align with the information provided in the previous paragraph.*

Thank you for this suggestion. The sentence has been updated as follows:

“Given the well-established link between CRP and systemic inflammation, the persistence of OC-induced CRP elevations in lean and highly active athletes may suggest that PA and low adiposity are insufficient for mitigating systemic inflammation induced by OCs.”

*3) Line 90: can the authors check and clarify if it is ‘both’ systemic inflammation and localised effects or is it ‘either’?*

Thank you for identifying this lack of clarity. The intended meaning of the statement is best reflected by the use of the word “or,” so the sentence has been updated as follows:

“There are several mechanisms by which OCs could increase CRP [17], which may include systemic inflammation or localized effects on hepatic production of CRP.”
 *4) When referring to synthetic estrogen the authors may consider using ethylestradiol vs estradiol (lines 91 ,94). This change in terminology may also be considered and edited throughout the paper.*

Thank you for prompting more careful consideration of this terminology. We believe it’s important for this terminology to provide as much specificity as possible without sacrificing accuracy. We have carefully revised the manuscript to maximize the specificity of our terminology regarding estrogens. When referring to the results of any intervention using a specific form of estrogen, we use the specific name of the form (i.e., ethinyl estradiol, estetrol, estradiol valerate, mestranol, etc.) used in the study. When referring to generalized statements that apply to all forms of currently used estrogens, or scenarios in which the exact form cannot be definitively ascertained, we use the generic term “estrogen,” with further specification (when necessary) as to whether we are referring to exogenous or endogenous estrogen.

*5) Line 113: I am not sure why the authors have selected their hypotheses based on OC- induced elevations not being associated with systemic inflammation. The information provided by the authors would suggest that the previous evidence not in favour of systemic inflammation is a) done in small homogenous samples (line 83) or is equivocal (line 96). The authors may need to consider a revision of their statement that justifies why hypothesis 2 was presented.*

We agree that the foundation for hypothesis 2 requires additional explanation and justification. We have added the following text to clarify:

“Given the well-established link between CRP and systemic inflammation, the persistence of OC-induced CRP elevations in lean and highly active athletes may suggest that PA and low adiposity are insufficient for mitigating systemic inflammation induced by OCs. Alternatively, these observations of high CRP levels in athletes may suggest that OC-induced CRP elevations are not reflective of systemic inflammation. In support of this concept, a randomized crossover trial by Van Rooijen et al. [16] previously reported significant CRP elevations without concomitant increases in interleukin-6 or tumor necrosis factor α in response to a combined OC containing ethinyl estradiol, which led the researchers to conclude that the observed CRP elevations were not indicative of a systemic inflammatory response.”
 *6) Line 155: should this statement include type and duration? While the authors have noted that less than 1% of US women may use progestin-only pills, there are different types (mono-,bi-, and tri-phasic OCPs) that women in the study may be using.*

Thank you for this suggestion. The section has been revised to improve clarity as follows:

“These NHANES questionnaires also provide incomplete information regarding the exact type, formulation, and generation of OC used by each study participant. As a result, the present analysis is not able to distinguish between progestin-only versus combined formulations, monophasic versus multiphasic formulations, different forms or generations of exogenous estrogen and progestin, or different dosages of exogenous estrogen and progestin.”

We have also updated the limitations section to acknowledge this point as concisely as possible. It now reads:

“These surveys do not identify the current menstrual cycle phase at the time of each participant’s NHANES assessment, but this unlikely to introduce a substantive or systematic bias to our analysis because CRP levels remain relatively stable across the menstrual cycle [24]. Surveys provided information about current OC use but did not provide details regarding duration of use or the exact type, formulation, and generation of OC used by each study participant. Nonetheless, these factors are unlikely to have a meaningful impact on our analysis. Crossover trials indicate that CRP elevations occur quickly during OC use and return to baseline quickly after cessation [16]. CRP responses are significantly attenuated by progestin-only formulations in comparison to combined formulations [19], but less than 1% of US women use progestin-only pills [39]. CRP responses do not appear to meaningfully differ when comparing among second, third, or fourth generation OCs [22], among active versus inactive pill phases [24], or among monophasic versus multiphasic formulations [40].”
 *7) Line 165: can the authors confirm if this statement of menstrual cycle status was in the later 4 years of the NHANES data? The previous section outlines differences between the data collection periods and the information here would be very helpful to the reader.*

Thank you for prompting this clarification. The content of this survey item remained unchanged from 1999-2006, but the identification number of the survey item was changed from RHQ050 to RHQ051 in 2003. The texted has been updated to clarify this change in numbering:

“Menstrual status was assessed by a survey item that asked participants, ‘When did you have your last period?’ This survey item was identified as RHQ050 from 1999-2002 and as RHQ051 from 2003-2006.”
 *8) Why is information on HRT presented between lines 170-173? Would it not be better suited to the paragraph above which specifically focuses on OCs data collection from the NHANES survey?*

Thank you for this suggestion. We have moved this information about HRT to the prior paragraph as directed.
 *9) Line 177: will need to be edited and have ‘menstrual cycle phases removed’. If an individual is using an OC then they do not have a menstrual cycle they will only have active and pill-free phases.*

Thank you for bringing this edit to our attention. It has been implemented as directed.
 *10) Physical activity’s abbreviation was defined in the abstract, however, there is inconsistent use of this abbreviation throughout the article. The authors may need to review this. Lines where the abbreviation has not been used have been identified: 86, 387, 391, 401, 411, and 490.*

Thank you for identifying this oversight. We have checked and corrected the article to ensure that all abbreviations have been used consistently throughout the manuscript.

**Reviewer #2**
*1) The authors have made all suggested comments and the manuscript has been significantly improved as a result, well done. This paper will hopefully prompt further research into the implications of elevated CRP concentrations amongst OC users - an important topic given the high prevalence of OC use amongst women.*

Thank you for your positive and constructive feedback throughout the review process.
